# An Unusual and Rare Case of a Seronegative Male Patient With Lupus Nephritis

**DOI:** 10.7759/cureus.9739

**Published:** 2020-08-14

**Authors:** Jeff Chow, Maureen N Achuko, Xiao-Lan Chen

**Affiliations:** 1 Internal Medicine, Cape Fear Valley Medical Center, Fayetteville, USA; 2 Nephrology, Cape Fear Valley Medical Center, Fayetteville, USA; 3 Pathology, Cape Fear Valley Medical Center, Fayetteville, USA

**Keywords:** seronegative lupus, sle, ana negative, race, ethnicity, lupus nephritis

## Abstract

Systemic lupus erythematosus (SLE) is an autoimmune disease that can affect nearly every organ system. The 2019 European League Against Rheumatism and American College of Rheumatology (EULAR/ACR) SLE have proposed an additive, weighted multicriteria system for classifying SLE while using antinuclear antibody (ANA) as an entry criteria. Most patients with SLE will test positive for ANA. We report a 28-year-old half Caucasian and half Asian male patient who initially presented with malar rash, severe bilateral lower extremity edema, and proteinuria. Typical serological tests associated with SLE were negative. The diagnosis of seronegative lupus nephritis was made with kidney biopsy that revealed class IV and V lupus nephritis. Creatinine improved from 2.06 to 0.87 mg/dL with oral mycophenolate mofetil (MMF) and a tapered dose of glucocorticoids. This case highlights the difficulty of diagnosis and treatment of seronegative lupus nephritis.

## Introduction

Systemic lupus erythematosus (SLE) is an autoimmune disease that can affect nearly every organ system. It is characterized by autoantibodies produced from an exaggerated response by B and T cells. The autoantibodies attach to various nuclear and cytoplasmic antigens, leading to end organ damage [1].

The diagnosis of SLE is based on clinical manifestations and laboratory findings. The 1982 revised American College of Rheumatology (ACR) SLE classification criteria have been used worldwide. The presence of at least four of 11 criteria is required for diagnosis. Since then, our understanding of the disease has advanced. Immunologic tests, such as low levels of serum complement components of C3 and C4, have been incorporated into clinical practice [2]. The antinuclear antibody (ANA) is often used as a screening test due to high sensitivity for autoimmune conditions. It plays a major immunopathogenic role in the disease. However, there is a small subset of patients, up to 5%, who test negative for ANA but have clinical features consistent with SLE [3]. Renal involvement can be clinically apparent in up to 50% of patients with SLE [1, 4]. Having a diagnosis of lupus nephritis is associated with a poor prognosis due to a higher risk for organ failure [5]. 

There have been reported cases of patients diagnosed with SLE despite negative ANA serological testing [3, 6]. In this report, we highlight a rare and complex case of a biracial, male patient who presented with lupus nephritis and subsequently diagnosed with seronegative SLE. 

## Case presentation

A 28-year-old half Caucasian and half Asian male presented to the ED with a three-month history of bilateral lower extremities swelling and an erythematous facial rash. He had associated symptoms of photosensitivity. Review of systems was negative for fever, infections, oral ulcers, and arthralgia. Past medical history was unremarkable. Family history was negative for kidney disease, autoimmune conditions, and diabetes mellitus.

**Figure 1:**
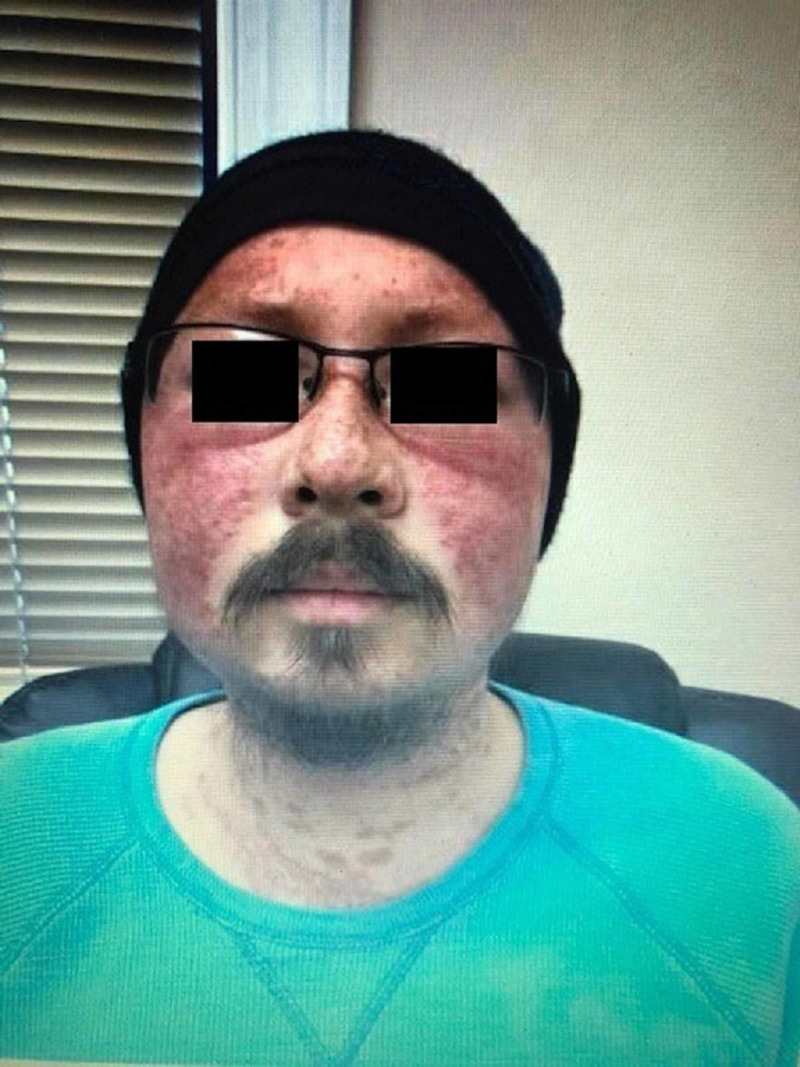
Illustration of erythematous malar rash. Eyes have been blotted out for patient de-identification.

On physical examination, blood pressure was 159/115 mmHg and temperature was 98 degree Fahrenheit. An erythematous rash was noted over the nose and across the middle of his cheeks (Figure 1). The upper chest also had erythematous maculopapular lesions. Bilateral lower extremity edema was present. 

Initial laboratory investigation revealed a hemoglobin of 16.7 g/dL and creatinine of 0.88 mg/dL. Urinalysis revealed 3+ protein and no red blood cells. C3 and C4 complement levels were low at 53 and 12 mg/dL respectively. Random urine protein and urine creatinine were 1418.8 and 135.8 mg/dL respectively. Random urine protein to urine creatinine was 10448 mg/g. ANA was negative. 

The patient was discharged from the ED and referred to a local nephrology clinic for further evaluation and management of nephrotic syndrome. Medications on discharge included candesartan 8 mg and torsemide 20 mg prescribed daily. The patient did not follow up at the nephrology clinic. 

Five months later, the patient showed up to the ED with worsening symptoms. The malar rash was still present. Bilateral pedal edema was 3+ and extended up to the lower abdomen. Laboratory investigation revealed hemoglobin of 9.4 g/dL. Comprehensive metabolic panel revealed creatinine of 2.06 mg/dL, blood urea nitrogen of 32 mg/dL, and albumin of 0.6 g/dL. Random urine protein and urine creatinine were 1121.6 and 93.10 mg/dL respectively. Random urine protein to urine creatinine was 12047 mg/g. HIV, lupus anticoagulant, ANA, antineutrophilic cytoplasmic antibody, double strand DNA, Smith, hepatitis C, hepatitis B, and anti-glomerular basement membrane were all negative. Renal ultrasound showed no evidence of hydronephrosis. A percutaneous CT guided kidney biopsy was performed. He received IV methylprednisolone and was tapered to oral prednisone 60 mg for three days. He was discharged on oral prednisone 40 mg daily and was recommended to follow up at the nephrology clinic for results of renal biopsy.

**Figure 2:**
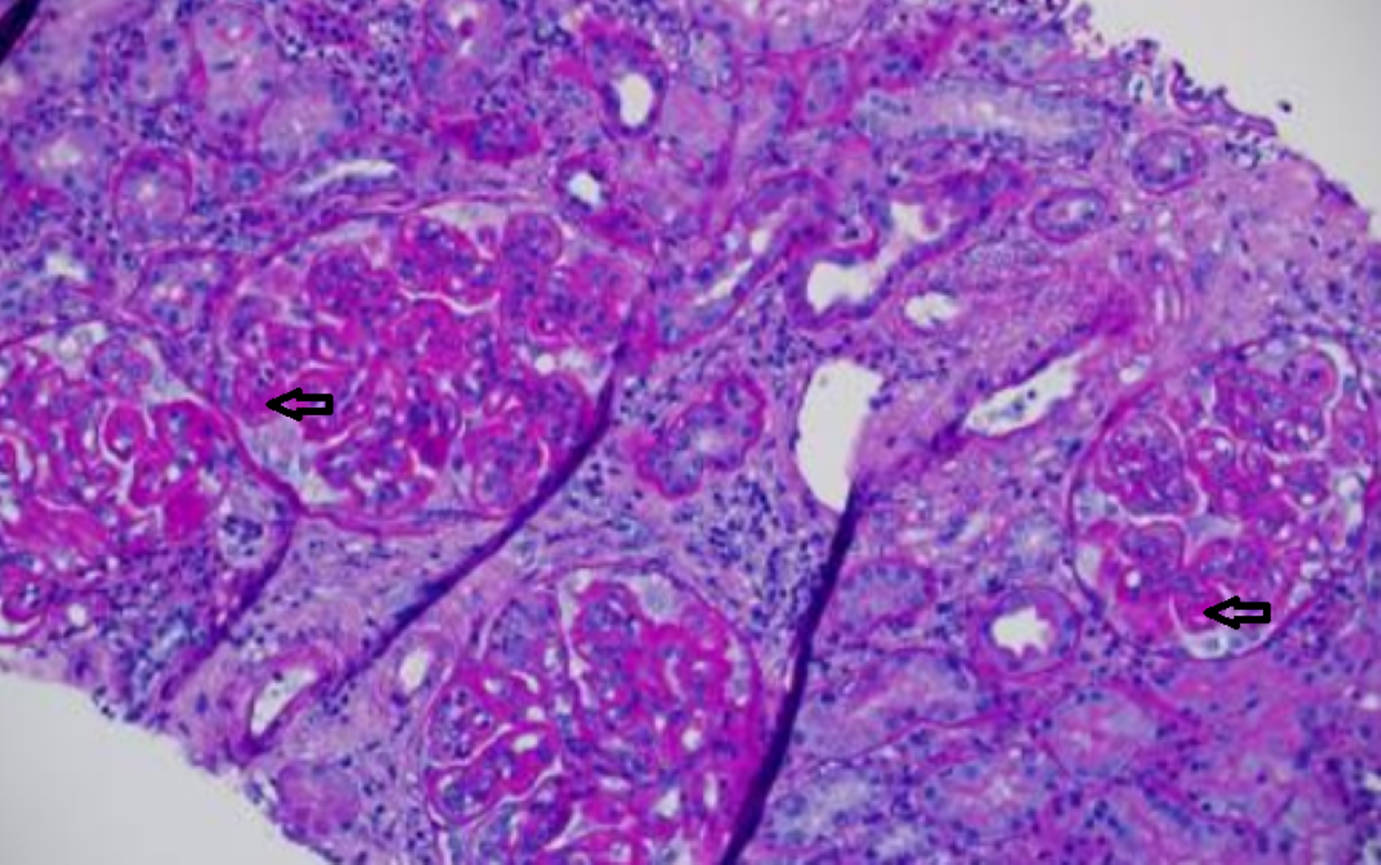
Light microscopy photo illustrates global and segmental proliferative and membranous glomerulonephritis with thickened capillary walls (arrows) (PAS stain, 200x).

**Figure 3:**
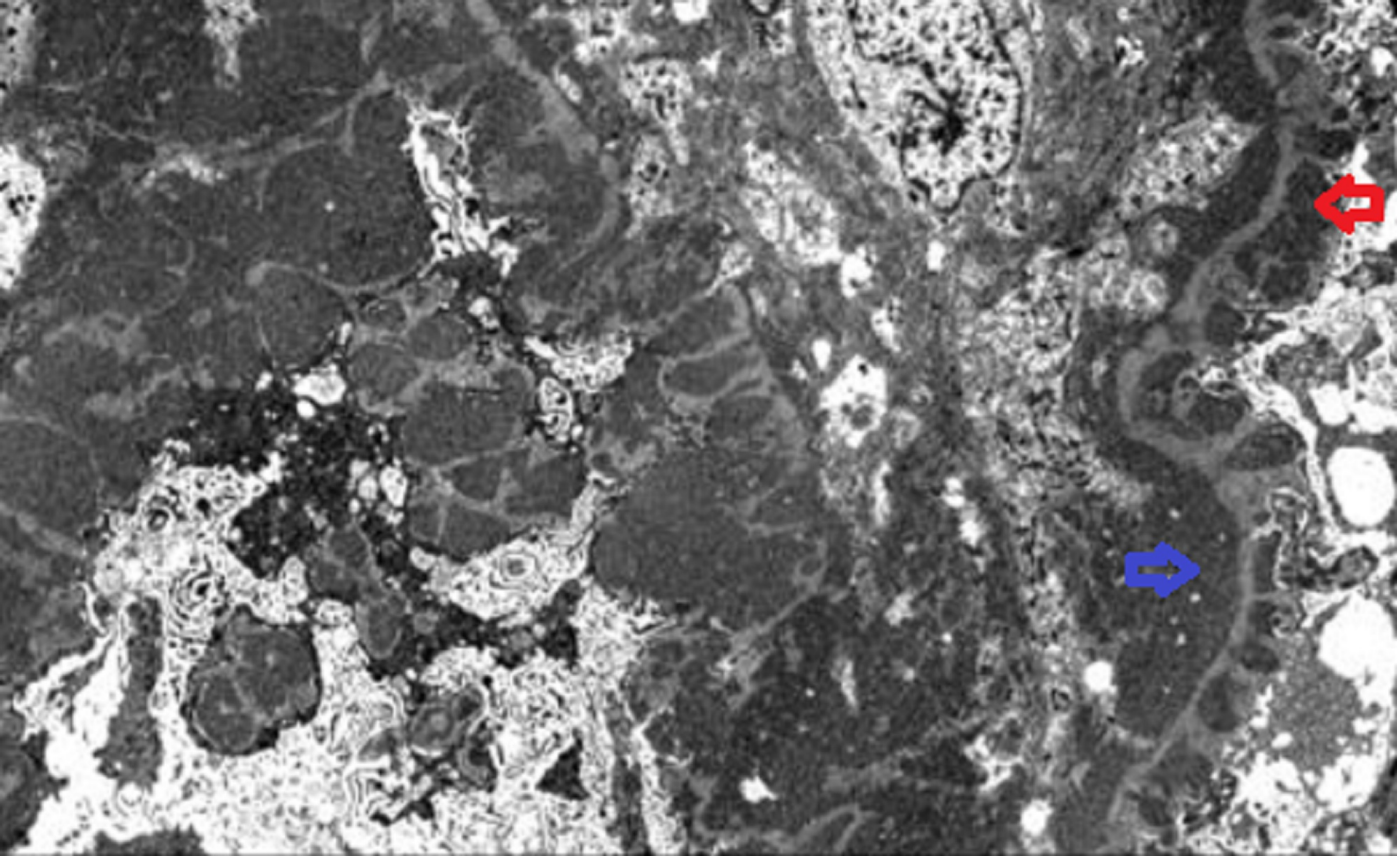
Electron microscopy photo illustrating a thickened basement membrane due to subepithelial (red arrow) and subendothelial (blue arrow) deposits.

The patient followed up at the nephrology clinic several days later. Kidney biopsy was indicative of a combined diffuse proliferative (type IV) and membranous (type V) lupus nephritis (Figures 2-3). 

He was diagnosed with ANA-negative lupus nephritis and treated with mycophenolate 750 mg twice a day and a tapered dose of oral steroids. Mycophenolate was increased to 1500 mg twice a day after two weeks. 

Three months later, the patient followed up in clinic. Bilateral lower extremity edema and facial resolved significantly. Random urine protein and urine creatinine were 839.8 and 170.9 mg/dL respectively. Random protein to creatinine ratio was 4914 mg/g. Creatinine improved to 0.87 mg/dL.

## Discussion

Characteristic manifestations of SLE include nonerosive arthritis of peripheral joints, oral ulcers, and malar rash [1]. This constellation will prompt clinicians to suspect SLE and investigate further with serological testing. Several investigators have reported a small group of SLE patients who are ANA negative. De Zosya et al. reported a patient presenting with pyrexia of unknown origin with renal and cerebral involvement who was diagnosed with seronegative SLE. Although helpful in diagnosis, reactive ANA and DsDNA antibody testing is not essential for SLE diagnosis [6]. 

Given the wide heterogeneity of clinical presentations of SLE, clinicians have relied on classification criteria to help make the diagnosis. Aringer et al. reported a new, weighted multicriteria system for SLE classification supported by the European League Against Rheumatism (EULAR) and ACR that use ANA as an entry criteria [2, 7]. The new classification has high sensitivity and specificity for SLE. In the article, Aringer et al. astutely pointed out that this new criteria cannot classify patients who are ANA negative and stresses that this particular subgroup of patients should not be excluded from SLE diagnosis and treatment [2]. Our patient falls into this subset. Serological workup for secondary causes of nephrotic syndrome was negative. A kidney biopsy was pursued to assist with diagnosis for clinical manifestations suggestive of lupus in the setting of equivocal autoimmune testing. The kidney biopsy showed type IV and V lupus nephritis. 

 Serological tests have greatly improved the accuracy of diagnosing lupus nephritis. However, patients with SLE who lack abnormal immunological serology are at risk for delayed diagnosis. The literature on seronegative lupus nephritis is limited and reporting of serologies for cases of SLE are variable because there is no standard immunological testing. Simmons et al. performed a review and found cases of lupus nephritis patients without positive SLE serology on initial presentation. Out of the 17 cases, seven developed positive serologies after treatment, although conversion of serology may take up to 10 years [8]. Serological testing can be misleading in these cases and puts patients at higher risk for progression to end stage renal disease. In these instances, kidney biopsy is critical for definitive diagnosis of lupus nephritis. 

Mycophenolate mofetil (MMF) has emerged as an immunosuppressive therapy in lupus nephritis that has comparable efficacy to cyclophosphamide. Appel et al. tested the efficacy of oral MMF versus intravenous cyclophosphamide (IVC) in a racially diverse population of patients with lupus nephritis and found that although MMF did not show superiority over IVC for induction of therapy, these drugs in combination with prednisone have similar efficacy in short-term induction therapy [9]. There is also evidence to suggest that outcomes with immunosuppressive therapy is influenced by race and ethnicity. There was no difference in response to MMF and IVC between Asian and White patients, but Black and Hispanic patients responded better to MMF than IVC [9]. Our patient racially identified himself as half Asian and half Caucasian. Renal function has improved while using oral MMF. 

## Conclusions

In conclusion, we report a case of a clinical dilemma of a male patient presenting with malar rash, photosensitivity, and proteinuria with negative serologies for SLE. Patients with ANA-negative lupus nephritis may not be classified under the new SLE classification system but this subset of patients should not be excluded from appropriate treatment. The kidney biopsy showed mixed diffuse proliferative and membranous pattern for lupus nephritis. IVC is the current standard treatment for lupus nephritis, but oral MMF has been found to have similar efficacy. Lupus nephritis can present with negative SLE antibodies and should be promptly diagnosed and treated.
